# Difference in resistance to humidity between commonly used dry powder inhalers: an *in vitro* study

**DOI:** 10.1038/npjpcrm.2016.53

**Published:** 2016-11-17

**Authors:** Christer Janson, Thomas Lööf, Gunilla Telg, Georgios Stratelis, Folke Nilsson

**Affiliations:** 1 Department of Medical Sciences: Respiratory, Allergy and Sleep Research, Uppsala University, Uppsala, Sweden; 2 AstraZeneca R&D, Mölndal, Sweden; 3 AstraZeneca Nordic-Baltic, Södertälje, Sweden; 4 Närhälsan Primary care, Kungshamn, Sweden

## Abstract

Multi-dose dry powder inhalers (DPIs) are commonly used in asthma and chronic obstructive lung disease (COPD) treatment. A disadvantage is their sensitivity to humidity. In real life, DPIs are periodically exposed to humid conditions, which may affect aerosol characteristics and lung deposition. This study compared DPI aerosol performance after exposure to humidity. Budesonide (BUD) inhalers (Turbuhaler; Novolizer; Easyhaler) and budesonide/formoterol (BUD/FORM) inhalers (Turbuhaler; Spiromax; Easyhaler) were stored in 75% relative humidity (RH) at both ambient temperature and at −0 °C. Delivered dose (DD) and fine-particle dose (FPD) were tested *in vitro* before and after storage. BUD inhalers: Turbuhaler and Novolizer showed only small decreases (<15%) in FPD in 40 °C/75% RH, whereas FPD for Easyhaler decreased by >60% (*P*=0.01) after 1.5 months of storage. Easyhaler also decreased significantly after 6 months of storage in ambient/75%RH by 25% and 54% for DD and FPD, respectively, whereas only small decreases were seen for Turbuhaler and Novolizer (<15%). BUD/FORM inhalers: Turbuhaler and Spiromax DD were unchanged in 40 °C/75% RH, whereas Easyhaler showed a small decrease. FPD (budesonide) decreased for Turbuhaler, Spiromax and Easyhaler by 18%, 10% and 68% (all significant), respectively, at 40 °C/75% RH. In ambient/75%RH, DD was unchanged for all inhalers, whereas FPD (budesonide) decreased for Spiromax (7%, *P*=0.02) and Easyhaler (34%, (*P*<0.01)). There are significant differences in device performance after exposure to humid conditions. A clinically relevant decrease of more than half FPD was seen for one of the inhalers, a decrease that may affect patients’ clinical outcomes. Prescriber and patient knowledge on device attributes are essential to ensure optimal drug delivery to the lungs.

## Introduction

Fixed or free combinations of inhaled corticosteroids (ICS) and long-acting β_2_-agonists are medications used in the treatment of asthma and chronic obstructive lung disease (COPD).^1^ A number of different devices are used for the actual drug delivery to the lungs—e.g., dry powder inhalers (DPIs) and pressurised metred dose inhalers. DPIs are commonly used in the Nordic countries. The major advantage of DPIs compared with pressurised metred dose inhalers is their general ease of use. However, an inherent disadvantage is their potential sensitivity to humidity at storage. Protection from the external environment to prevent moisture uptake is necessary,^2^ which is why different manufacturers use different humidity protections to prolong shelf life.

Data regarding inhaler humidity resistance are scarcely available in the public domain. *In vitro* studies have shown that the actual delivered dose may be negatively affected by storage in humid conditions.^3,4^ In a comparison of two commonly used DPIs, fine-particle doses (FPD) for budesonide (BUD)/formoterol (FORM) Turbuhaler were virtually unchanged after 3 months of storage in 40 °C/75% relative humidity (RH), whereas the FPD for fluticasone/salmeterol Diskus decreased by about 50% even though there was no decrease in delivered dose (DD).^4^ Furthermore, a recent study showed that another DPI, Spiromax, appeared to be unaffected when tested at high humidity.^5^

The past years have witnessed an increased number of DPIs available on the market, both branded and analogue products. Analogues are considered bioequivalent if they are pharmaceutically equivalent or show bioavailability within the acceptable limits between ⩾80.00% and ⩽125.00%.^6^ However, different devices have different instructions regarding storage, time in-use, dose loading and inhaler technique. Suboptimal inhaler technique^7^ and inhaler mishandling^8^ are still common in real-life use, and lack of device continuity may increase these problems.^9,10^ By the availability of many new products, using different devices to deliver drugs to the lungs, asthma and COPD patients are likely to be exposed to more than one type of inhaler during their treatment journey, potentially also concurrently.

In real-life use, DPIs are likely to be exposed to humid conditions, which may affect aerosol characteristics and lung deposition. If thorough instructions are not given, the risk of mishandling is evident. Therefore, patient education is increasingly important to ensure optimal inhaler use.^11^

The present study compared DPI aerosol performance after exposure to 75% humidity in ambient and accelerated temperature for commonly used DPIs containing BUD alone or BUD/FORM in a fixed combination.

## Results

### Single BUD inhalers

#### 40 °C/75% RH

For inhalers stored at 40 °C/75% RH, there was a small decrease (<15%) in FPD for both the Turbuhaler and Novolizer, whereas the decrease in BUD FPD for Easyhaler was more than 60% after 1.5 months, after which it did not change significantly over time. BUD DD for Easyhaler continued to decrease beyond the 1.5-month observation, and the total observed change was almost 40% (Table 1).

#### Ambient temperature/75% RH

**Figure 1:**
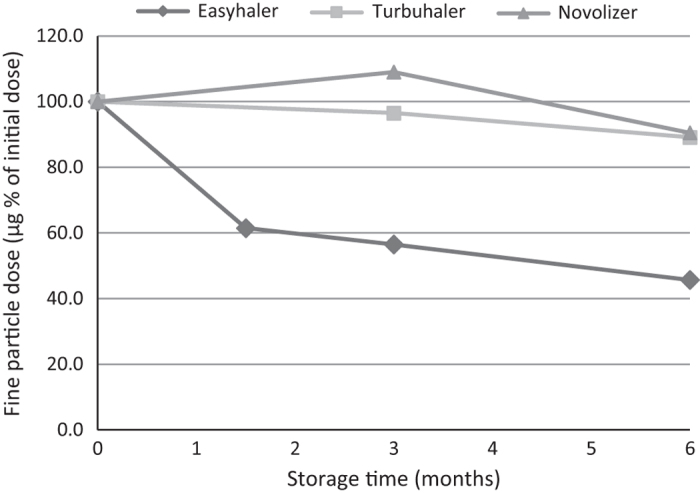
Single budesonide inhalers. Fine-particle dose after storage at ambient temperature and 75% relative humidity.

For inhalers stored in ambient/75% RH, there was no statistically significant decrease in the respective DD and FPD after 6 months of storage for Turbuhaler (8 and 11%). For Novolizer, DD decreased by 14% (statistically significant) and FPD decreased by 10% (non-significant). For Easyhaler, the BUD DD decreased by 25% and the FPD decreased by 54% (both statistically significant), a decrease in FPD that was most obvious during the first 1.5 months of storage (Table 1, Figure 1).

### Fixed-combination BUD/FORM inhalers

#### 40 °C/75% RH

For inhalers stored at 40 °C/75% RH, there were no statistically significant changes in DD for either BUD or FORM for Turbuhaler and Spiromax; a small non-significant decrease was seen for Easyhaler. A decrease in BUD FPD was observed for all three inhalers after 3 months (Table 2), with 18% for Turbuhaler, 10% for Spiromax and 68% for Easyhaler. For the Easyhaler, a decline in FORM FPD of 48% was observed (Table 2).

#### Ambient temperature/75% RH

**Figure 2:**
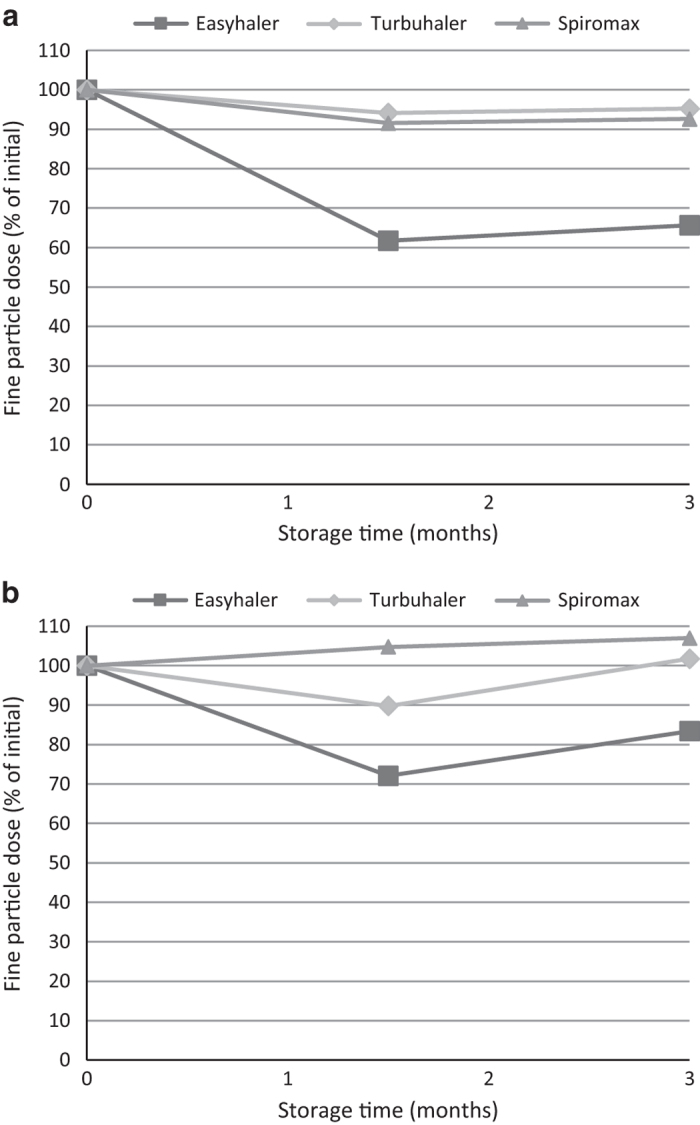
Fixed-combination budesonide/formoterol inhalers. (**a**) Fine-particle dose budesonide, and (**b**) fine-particle dose formoterol after storage at ambient temperature and 75% relative humidity.

For inhalers stored in ambient/75% RH, both BUD and FORM DD were virtually unchanged for all tested inhalers. For BUD FPD, a slight decrease was noted after 3 months of storage for Turbuhaler and Spiromax (5% and 7%, respectively). Easyhaler declined in BUD FPD by 34%, a decrease that was observed already after 1.5 months of storage (Table 2, Figure 2). Further, a decrease of 20% was found for Easyhaler in FORM FPD, whereas no change was found for the other two inhalers (Table 2).

## Discussion

### Main findings

This *in vitro* study examined the performance of a number of commonly used multi-dose DPIs when stored under humid conditions in ambient and elevated temperature. The study showed that different inhalers have different resistances to humidity, affecting both DD and FPD. One of the examined inhalers had a pronounced sensitivity to humidity, resulting in a significant and clinically relevant decrease in FPD. The other inhalers tested did not show any clinically relevant decrease in FPD after storage.

### Interpretation of findings in relation to previously published work

There may be a number of reasons for the observed findings in our study. Although most of the examined inhalers have virtually no moisture protection once the protective overwrap has been removed, the Turbuhaler has an inherent moisture protection, as it contains a desiccant and has a tight cover that screws tightly to the turning grip. The influence of moisture on formulations using ordered mixtures with coarse lactose carrier particles may be another reason for the differences seen. It has previously been suggested that the mechanism of decreased dispersibility of Active Pharmaceutical Ingredient particles from a lactose carrier was related to increased capillary/solid bridging interactions due to increased polar surface energy,^12^ which increased during storage at high humidity. However, it was also concluded that the process of dispersion is very complicated and that surface energy is not the only important factor. An ideal lactose carrier blend for DPIs includes drug particles and lactose carrier particles with a high degree of crystallinity, with no or very low amounts of amorphous content on the particle surfaces.^13^ The reason for this is to ensure a consistent particle–particle interaction—i.e., a consistent FPD when inhaled, also after exposure to heat and/or elevated humidity. When exposed to moisture, amorphous material will start to recrystallise; the kinetics (time frame) for this is dependent on both temperature and humidity, but also on how easily the water molecules can reach all surfaces of the lactose particles. The process may start already at room temperature at relatively low RH levels—e.g., 35% RH.^14,15^ The complexity in the dispersion process is illustrated by the fact that both Novolizer and Spiromax, which contain ordered mixtures, show a significantly better resistance to humidity compared with the Easyhaler, which also contains an ordered mixture. It could be speculated that one reason for the differences observed in this study is because of the inherent properties of the different types of lactose carriers used—e.g., containing different levels of amorphicity—how the carriers are prepared before mixing or how the mixing of the drug particles with the carrier particles is carried out for the different products.

In the inhaled treatment of asthma and COPD, the clinical effect is related to the amount of inhaled drug that reaches the lungs, which depends on the amount of fine particles generated at inhalation. Aerodynamic particle size has a major impact on the regional lung deposition. Most large particles (>5 μm) are deposited in the oropharyngeal and central airways, whereas smaller particles (<5 μm) are likely to pass through the upper regions of the lungs and are deposited in the bronchioles.^16,17^ In the paper by Labiris *et al*., the β_2_-agonist salbutamol produced a larger bronchodilator response at all doses with the small-particle aerosol compared with the larger particle size aerosol.^18^ This may influence the efficacy and safety of inhaled drugs.^16,17,19^

Optimally, the FPD should be stable over time, irrespective of storage conditions. Previous studies have indicated that asthma medications, despite intraclass similarities, should not be considered as interchangeable.^9,20–22^ Switching a patient from one type of device to another without education may result in confusion regarding the new device handling, with more critical handling errors leading to decreased dose delivery.^21,23–25^ The difference in humidity resistance observed in our study is adding to this complexity as one more factor that should be taken into account.

### Strengths and limitations of this study

This study is not without limitations, the most important being that it has been conducted *in vitro.* The inhalers were not tested after everyday patient use with potential impact by additional real-life conditions (e.g., different device handling errors, dropping, low flow rates or other patient-related factors), which may further have affected the results. The samples were stored in 75% RH (a condition frequently used in stability studies of pharmaceutical drug products; International Conference on Harmonisation of Technical Requirements for Registration of pharmaceuticals for Human Use: ICH Harmonised Tripartite Guideline, Stability Testing of New Drug Substances and Products Q1A(R2), 2003) with two different temperatures: one ambient and one accelerated temperature. A 75% RH is not an extreme value, as values close to 100% RH can be measured in a bathroom or a kitchen, where patients commonly store their medications,^22^ and the RH can be much higher in many countries during part of the year.^4^ In addition, the indoor humidity (in Sweden recommended to be between 30 and 70% RH) has, as a consequence of energy-saving efforts, been reported to exceed the recommended levels.^26,27^ However, the relevance of the accelerated conditions (40 °C/75% RH) in real life may be discussed, as it represents a temperature that is higher than normal temperature in most countries in the northern part of the world.

The clinical implications associated with the findings in this *in vitro* study have not been investigated. However, a previous report has confirmed the *in vitro* findings with an *in vivo* part of the same study, where it was shown that actual lung deposition was reduced in the same magnitude as the observed FPD decline seen *in vitro.*^4^ Applying the *in vivo* findings in that study, it may be that the observed significant reduction of FPD seen for one of the inhalers in the present study would be followed by an equivalent decline in lung deposition, with potential implications for the clinical outcome. Further studies would be required to investigate this.

### Implications for future research, policy and practice

Asthma and COPD are diseases associated with a considerable burden on healthcare budgets, and cost-containment measures are increasing as less costly devices become available.^28,29^ All inhalers are not the same, and this may constitute a challenge, not only when considering cost-effectiveness analyses but also regarding device handling. It has been shown that healthcare professionals, including staff from the pulmonary department, have limited knowledge regarding the handling of the most commonly used devices.^30^ In addition, patients may have inhalers switched without consultation, something that was shown in a recent observational matched cohort study in general practice in Sweden, investigating device switching in an asthma population.^29^ In that study, 54% of the patients were switched without a primary care visit, indicating switch without training, which was associated with 25% more exacerbations and 148% more outpatient hospital visits compared with non-switchers.^29^ It is thus vital to ensure that healthcare providers have sufficient knowledge of all the different inhalers’ attributes and that they can perform patient educational interventions in optimal device handling and storage.

### Conclusion

This study shows that there are significant differences in device performance after exposure to slightly increased humid conditions for one of the inhalers tested. These differences are not likely to be known, but with the increasing number of devices available on the market knowledge of different device attributes is crucial to ensure a safe and effective drug delivery to the lungs. In real life, it is likely that inhalers are exposed to humid conditions, affecting the performance of the device. Further studies may be needed to investigate whether the marked decrease observed for one of the inhalers in this study causes significant effect on patients’ clinical outcomes.

## Materials and methods

### Materials

#### *In vitro* tests

Single BUD inhalers (200 μg per dose, 200 doses). The following BUD single inhalers were tested: Turbuhaler (Pulmicort Turbuhaler, AstraZeneca, Södertälje, Sweden); Novolizer (Novopulmon Novolizer, Meda, Solna, Sweden); and Easyhaler (Giona Easyhaler, Orion Pharma, Espoo, Finland). For each product, the same batch was used throughout the study. Both Novolizer and Easyhaler are protected from humidity by an aluminium overwrap, and the in-use shelf life is 6 months for both products after removing the protective package. Turbuhaler has an inherent moisture protection, as it contains a desiccant and has a tight cover that screws tightly to the turning grip.

#### Fixed-combinations BUD/FORM inhalers (160/4.5 μg per dose, 120 doses)

The following fixed combinations of BUD/FORM inhalers were tested: Turbuhaler (Symbicort Turbuhaler, AstraZeneca, Södertälje, Sweden), Spiromax (DuoResp Spiromax, TEVA Pharmaceutical, Petach Tikva, Israel) and Easyhaler (Bufomix Easyhaler, Orion Pharma, Espoo Finland). For each product, the same batch was used throughout the study. Spiromax is protected from humidity by an aluminium overwrap, and the in-use shelf life is 6 months after removing the protective package. Easyhaler and Turbuhaler are described above.

#### Settings

All inhalers had at least 10 months of shelf-life time left at the study start (initiation of storage), and their performance was tested/examined before the end of shelf life. A decrease of >20% in DD and FPD *in vitro* was predefined as clinically relevant based on the bioequivalence demands from European Medicines Agency.^6^

### Storage conditions

The inhalers were stored at ambient temperature (measured to be between 19 and 22 °C) and 75% controlled RH, as well as at 40 °C/75% RH (controlled accelerated condition, from agreed standard stability testing conditions as given in the International Conference on Harmonization (ICH) stability guideline) (Ref: CPMP/QWP/609/96/Rev 2, 2007) (International Conference on Harmonisation of Technical Requirements for Registration of pharmaceuticals for Human Use: ICH Harmonised Tripartite Guideline, Stability Testing of New Drug Substances and Products Q1A(R2), 2003).

The inhalers were stored according to the patient information leaflet during the in-use time. Thus, Turbuhaler was stored with the dust- and moisture-protective cover, whereas Easyhaler, Novolizer and Spiromax were stored without the protective overwrap, but with their dust caps on the mouthpiece.

In the single BUD inhaler test, five inhalers per product were tested at the initial time point and after 6 months, whereas three inhalers per product were tested at the intermediate time points. Testing after 1.5 months of storage in ambient temperature/75% RH for Turbuhaler and Novolizer samples was omitted because the changes observed after 1.5 months of storage at the accelerated condition, 40 °C/75% RH, were non-significant (Table 1) and thereby not likely to be significant in the non-accelerated condition (standard drug product stability testing).

In the fixed-combinations BUD/FORM test, five inhalers per product were tested at all time points, and the 6-month measurement was omitted based on the learnings from the single BUD inhaler test where it was observed that most of the changes occurred already during the first 3 months.

The *in vitro* study was performed by Emmace Consulting AB (Lund, Sweden). The testing was performed sequentially.

### Cascade impactor analysis

The DD and the FPD were measured before storage (baseline), after 1.5 months and at 3 months and 6 months (single inhaler test only) of storage both at ambient/75% RH and at 40 °C/75% RH, using the Next-Generation Impactor operating at a fixed pressure drop of 4 kPa (ref. 31; United States Pharmacopeia: USP38, Chapter 601: Physical tests and determinations; Inhalation and Nasal Drug Products: 410-413.). DD is defined as the total amount of Active Pharmaceutical Ingredient delivered to the Next-Generation Impactor. FPD is defined as the amount of Active Pharmaceutical Ingredient contained in particles <5 μm in size, and the amount was calculated using interpolation between relevant stages, which depend on the flow rate (stage cutoffs vary with varying flow rate). The volume of air drawn through the inhalers and the Next-Generation Impactor was fixed to 4 l. For each product, storage condition and time point, 6 doses were tested.

### Quantitative analysis

BUD and BUD/FORM were quantified using validated liquid chromatographic methods with ultraviolet detection.

### Statistical analysis

For each product, storage condition and time point, the effects of storage were assessed by comparing the results against the initial values using a paired *t*-test. Bonferroni’s correction for multiple tests was applied^32^ to declare statistical significance with an overall *α* level of 0.05 (Svensk Standard SS-EN ISO 7730).

## Figures and Tables

**Table 1:** DD and FPD after storage of single BUD inhaler in 75% RH at both ambient temperature and at 40 °C

*Parameter*	*Time*	*Turbuhaler*	P *value*	*Novolizer*	P *value*	*Easyhaler*	P *value*
*40 °C/75% RH*							
DD^a^ μg per dose	Baseline	138		192		198	
	1.5 months	119	0.12	165	0.04	151	0.01^b^
	3 months	150	0.33	193	0.94	168	0.07
	6 months	120	0.10	163	<0.01^b^	125	<0.01
FPD^c^ μg per dose	Baseline	74		78		54	
	1.5 months	66	0.19	71	0.15	20	<0.01^b^
	3 months	79	0.38	73	0.25	21	<0.01^b^
	6 months	63	0.04	68	0.02	20	<0.01^b^
							
*Ambient temp/75% RH*							
DD^a^ μg per dose	Baseline	138		192		198	
	1.5 months	NT^d^		NT^d^		170	0.10
	3 months	134	0.67	209	0.22	179	0.25
	6 months	127	0.24	165	0.01^e^	149	<0.01^b^
FPD^c^ μg per dose	Baseline	74		78		54	
	1.5 months	NT^d^		NT		33	<0.01^e^
	3 months	72	0.67	85	0.15	30	<0.01^e^
	6 months	66	0.11	71	0.06	25	<0.01^e^

Abbreviations: API, Active Pharmaceutical Ingredient; DD, delivered dose; FPD, fine-particle dose; NGI, Next-Generation Impactor; NT, not tested; RH, relative humidity.

a DD is defined as the total amount of API delivered to the NGI.

b Statistically significant, applying Bonferroni’s correction for three comparisons (*P*<0.0167=0.05/3).

c FPD is defined as the amount of API contained in particles <5 μm in size, and the amount was calculated using interpolation between relevant stages, which depend on the flow rate (stage cutoffs vary with varying flow rate).

d Only Giona Easyhaler was tested after storage for 1.5 months at ambient/75% RH, as this was a conditional test carried out only if significant changes were observed after storage at 40 °C/75% RH for 6 weeks.

e Statistically significant, applying Bonferroni’s correction for two comparisons (*P*<0.025=0.05/2).

**Table 2:** DD and FPD after storage of fixed-combination BUD/FORM inhalers in 75% RH at both ambient temperature and at 40 °C

*Parameter*	*Time*	*Turbuhaler*		P *value*	*Spiromax*		P *value*	*Easyhaler*		P *value*
		*BUD*	*FORM*	*BUD*	*BUD*	*FORM*	*BUD*	*BUD*	*FORM*	*BUD*
*40°/75% RH*										
DD^a^ μg per dose	Baseline	147	3.0		142	3.6		181	4.0	
	1.5 months	151	3.9	0.31	144	3.7	0.29	159	3.8	0.02^b^
	3 months	151	4.0	0.41	149	3.9	0.04	166	4.0	0.14
FPD^c^ μg per dose	Baseline	86	2.0		68	1.5		71	1.5	
	1.5 months	78	1.9	0.01^b^	61	1.4	0.03	29	0.8	<0.01^b^
	3 months	70	1.8	<0.01^b^	61	1.6	<0.01^b^	23	0.8	<0.01^b^
										
*Ambient temp/75% RH*										
DD^c^ μg per dose	Baseline	147	3.8		142	3.6		181	4.0	
	1.5 months	145	3.6	0.58	147	4.0	0.11	190	4.5	0.37
	3 months	154	4.1	0.07	143	4.0	0.63	172	4.3	0.34
FPD^c^ μg per dose	Baseline	86	2.0		68	1.5		71	1.5	
	1.5 months	81	1.8	0.16	62	1.6	<0.01^b^	44	1.1	<0.01^b^
	3 months	82	2.0	0.07	63	1.7	0.02^b^	47	1.2	<0.01^b^

Abbreviations: API, Active Pharmaceutical Ingredient; BUD, Budesonide; DD, delivered dose; FORM, formoterol; FPD, fine-particle dose; NGI, Next-Generation Impactor; RH, relative humidity.

a DD is defined as the total amount of API delivered to the NGI.

b Statistically significant, applying Bonferroni’s correction for two comparisons (*P*<0.025=0.05/2).

c FPD is defined as the amount of API contained in particles <5 μm in size, and the amount was calculated using interpolation between relevant stages, which depend on the flow rate (stage cutoffs vary with varying flow rate).

## References

[bib1] GINA report, Global Strategy for Asthma Management and Prevention (2014); Available at www.ginasthma.org. Accessed on 19 January 2015.

[bib2] Maggi, L., Bruni, R. & Conte, U. Influence of the moisture on the performance of a new dry powder inhaler. Int. J. Pharm. 177, 83–91 (1999). 10205605 10.1016/s0378-5173(98)00326-3

[bib3] Asking L., Axelsson M. & Lindberg J. Aluminium blisters may fail to protect against humidity. Drug Delivery to the Lungs IX. 84–87 (1998).

[bib4] Borgström, L., Asking, L. & Lipniunas, P. An in vivo and in vitro comparison of two powder inhalers following storage at hot/humid conditions. J. Aerosol MED. 18, 304–310 (2005). 16181005 10.1089/jam.2005.18.304

[bib5] Canonica, G. W., Arp, J., Keegstra, J. R. & Chrystyn, H. Spiromax, a new dry powder inhaler: dose consistency under simulated real-world conditions. J. Aerosol Med. Pulm/ Drug Deliv/ 28, 309–319 (2015). 10.1089/jamp.2015.1216 PMC4601554 26352860

[bib6] Guideline on the investigation of bioequivalence. Available at http://www.ema.europa.eu/docs/en_GB/document_library/Scientific_guideline/2010/01/WC500070039.pdf. Accessed on 12 March 2015.

[bib7] Papi, A. et al. Inhaler devices for asthma: a call for action in a neglected field. Eur. Respir. J. 37, 982–985 (2011). 21532013 10.1183/09031936.00150910

[bib8] Melani, A. S. et al. Inhaler mishandling remains common in real life and is associated with reduced disease control. Respir. Med. 105, 930–938 (2011). 21367593 10.1016/j.rmed.2011.01.005

[bib9] Lavorini, F. et al. Switching from branded to generic inhaled medications: potential impact on asthma and COPD. Expert Opin. Drug Deliv. 10, 1597–1602 (2013). 24224777 10.1517/17425247.2013.852182

[bib10] Bjermer, L. The importance of continuity in inhaler device choice for asthma and COPD. Respiration 88, 346–352 (2014). 25195762 10.1159/000363771

[bib11] Price, D. The way forward: dry powder inhalers should only be switched with physician agreement and patient training. Int. J. Clin. Pract. Suppl. 36–37 (2005). 16280003 10.1111/j.1368-504X.2005.00727.x

[bib12] Das, S., Larson, I., Young, P. & Stewart, P. Surface energy changes and their relationship with the dispersibility of salmeterol xinafoate powders for inhalation after storage at high RH. Eur. J. Pharm. Sci. 38, 347–354 (2009). 19732829 10.1016/j.ejps.2009.08.007

[bib13] Timmermann, I.-L., Steckel, H. & Trunk, M. Assessing, the re-crystallisation behaviour of amorphous lactose using the RH-perfusion cell. Eur. J. Pharm. Biopharm. 64, 107 (2006). 16527465 10.1016/j.ejpb.2006.01.011

[bib14] Elamin, A. A., Sebhatu, T. & Ahlneck, C. The use of amorphous model substances to study mechanically activated materials in the solid state. Int. J. Pharm. 119, 25–36 (1995).

[bib15] Briggner, L.-E. The use of isothermal microcalorimetry in the study of changes in crystallinity induced during the processing of powders. Int. J. Pharm. 105, 125–135 (1994).

[bib16] Lee, S. et al. In vitro considerations to support bioequivalence of locally acting drugs in dry powder inhalers for lung diseases. AAPS J. 11, 414–423 (2009). 19495991 10.1208/s12248-009-9121-4 PMC2758114

[bib17] Usmani, O. S., Biddiscombe, M. F. & Barnes, P. J. Regional lung deposition and bronchodilator response as a function of β2-agonist particle size. Am. J. Respir. Crit. Care Med. 172, 1497–1504 (2005). 16192448 10.1164/rccm.200410-1414OC

[bib18] Labiris et al. Pulmonary drug delivery. Part II: The role of inhalant delivery devices and drug formulations in therapeutic effectiveness of aerosolized medications. Br. J. Clin. Pharmacol. 56, 588–599 (2003). 14616419 10.1046/j.1365-2125.2003.01893.x PMC1884297

[bib19] Zanen, P., Go, L. T. & Lammers, J. W. Optimal particle size for beta 2 agonist and anticholinergic aerosols in patients with severe airflow obstruction. Thorax 51, 977–980 (1996). 8977595 10.1136/thx.51.10.977 PMC472643

[bib20] Doyle, S. et al. What happens to patients who have their asthma device switched without their consent? Prim. Care Respir. J. 19, 131–139 (2010). 20174771 10.4104/pcrj.2010.00009 PMC6602231

[bib21] Thomas, M. et al. Inhaled corticosteroids for asthma: impact of practice level device switching on asthma control. BMC Pulmon. Med. 9, 1 (2009). 10.1186/1471-2466-9-1 PMC2636760 19121204

[bib22] Price, D., Summers, M. & Zanen, P. Could interchangeable use of dry powder inhalers affect patients? Int. J. Clin. Pract. Suppl. Suppl 3–6 (2005). 10.1111/j.1368-504X.2005.00720.x 16279996

[bib23] Haughney, J. et al. Choosing inhaler devices for people with asthma: current knowledge and outstanding research needs. Respir. Med. 104, 1237–1245 (2010). 20472415 10.1016/j.rmed.2010.04.012

[bib24] Schulte, M. et al. Handling of and preferences for available dry powder inhaler systems by patients with asthma and COPD. J. Aerosol Med. Pulmon. Drug. Deliv. 21, 321–328 (2008). 10.1089/jamp.2007.0634 18823257

[bib25] Laube, B. L. et al. ERS/ISAM Task force report. What the pulmonary specialist should know about the new inhalation therapies. Eur. Respir. J. 37, 1308–1331 (2011). 21310878 10.1183/09031936.00166410

[bib26] Bornehag, C. G., Sundell, J., Hägerhed-Engman, L. & Sigsgaard, T. ‘Association between Ventilation Rates in 390 Swedish Homes and Allergic Symptoms in Children’, Indoor Air 15, 275–280 (2005). 10.1111/j.1600-0668.2005.00372.x 15982274

[bib27] Engvall K. & Norrby C. Sick building syndrome in relation to building dampness in multi-family residential buildings in Stockholm. Int. Arch. Occup. Environ. Health 74, 270–278. (2001). 11401019 10.1007/s004200000218

[bib28] Weiss, K. B. & Sullivan, S. D. The health economics of asthma and rhinitis. I. Assessing the economic impact. J. Allergy Clin. Immunol. 107, 3–8 (2001). 11149982 10.1067/mai.2001.112262

[bib29] Ekberg-Jansson A. et al. Budesonide inhaler device switch patterns in an asthma population in Swedish clinical practice (ASSURE). Int. J. Clin. Pract. 69: 1171–1178. (2015). 10.1111/ijcp.12685 26234385

[bib30] Grydeland, T. B., Methlie, P. & Bakke, P. S. Instructing patients in the correct use of inhalation devices (in Norweigian). Tidsskr. Nor Laegeforen 126, 312–314 (2006). 16440037

[bib31] European Directorate for Quality in Medicines and Healthcare Preparations for Inhalation: Aerodynamic Assessment of Fine Particles. 8th edn, 316–319, Ch. 2.9.18 (European Pharmacopeia, 2014).

[bib32] Bland, J. M. & Altman, D. G. Multiple significance tests: the Bonferroni method. BMJ 310, 170 (1995). 7833759 10.1136/bmj.310.6973.170 PMC2548561

